# Microbiome of root vegetables—a source of gluten-degrading bacteria

**DOI:** 10.1007/s00253-020-10852-0

**Published:** 2020-09-02

**Authors:** Viia Kõiv, Kaarel Adamberg, Signe Adamberg, Ingrid Sumeri, Sergo Kasvandik, Veljo Kisand, Ülo Maiväli, Tanel Tenson

**Affiliations:** 1 Institute of Technology, University of Tartu, Tartu, Estonia; 2 School of Science: Department of Chemistry and Biotechnology, Tallinn University of Technology, Tallinn, Estonia; 3 Center of Food and Fermentation Technologies, Tallinn, Estonia

**Keywords:** Prolyl endopeptidase, Celiac disease, ELISA, Food, Root vegetable

## Abstract

**Abstract:**

Gluten is a cereal protein that is incompletely digested by human proteolytic enzymes that create immunogenic peptides that accumulate in the gastrointestinal tract (GIT). Although both environmental and human bacteria have been shown to expedite gluten hydrolysis, gluten intolerance is a growing concern. Here we hypothesize that together with food, we acquire environmental bacteria that could impact our GIT with gluten-degrading bacteria. Using in vitro gastrointestinal simulation conditions, we evaluated the capacity of endophytic bacteria that inhabit root vegetables, potato (*Solanum tuberosum*), carrot (*Daucus sativus*), beet (*Beta vulgaris*), and topinambur (Jerusalem artichoke) (*Helianthus tuberosus*), to resist these conditions and degrade gluten. By 16S rDNA sequencing, we discovered that bacteria from the families *Enterobacteriaceae*, *Bacillaceae*, and *Clostridiaceae* most effectively multiply in conditions similar to the human GIT (microoxic conditions, 37 °C) while utilizing vegetable material and gluten as nutrients. Additionally, we used stomach simulation (1 h, pH 3) and intestinal simulation (1 h, bile salts 0.4%) treatments. The bacteria that survived this treatment retained the ability to degrade gluten epitopes but at lower levels. Four bacterial strains belonging to species *Bacillus pumilus*, *Clostridium subterminale*, and *Clostridium sporogenes* isolated from vegetable roots produced proteases with postproline cleaving activity that successfully neutralized the toxic immunogenic epitopes.

**Key points:**

• *Bacteria from root vegetables can degrade gluten.*

• *Some of these bacteria can resist conditions mimicking gastrointestinal tract.*

**Electronic supplementary material:**

The online version of this article (10.1007/s00253-020-10852-0) contains supplementary material, which is available to authorized users.

## Introduction

Gluten containing grains, especially wheat, are the main carbohydrate and plant protein sources in Western diets. Wheat is composed of 8–15% protein, from which 85–90% is gluten (Biesiekierski 2017). Gluten is a complex mixture of proteins, divided by the solubility in aqueous alcohols into two protein families: the gliadins and the glutenins. Gluten proteins have a unique primary amino acid structure and contain many glutamine (38%) and proline residues (20%) and repetitive PQ peptide sequences (Wieser 2007). Due to the structure of proline, human gastric and pancreatic enzymes do not efficiently cleave the peptide bonds of proline-rich proteins and generate pathogenic peptides, which contribute to three types of human disorders: autoimmune celiac disease (CD), allergy to wheat, and non-celiac gluten sensitivity (NCGS) (van De Wal et al. 1998; van de Wal et al. 1999; ArentzHansen et al. 2002). The prevalence of these three disorders has increased over the last two decades, which suggests an important environmental and/or lifestyle contribution to this susceptibility (Meijer et al. 2018).

The main therapy of gluten intolerance disorders is avoidance of wheat. Experimental strategies include reducing the immune response by modifying gluten in the diet of susceptible patients (Merz et al. 2016; Scherf et al. 2018) and enzyme therapy that supplements gluten-degrading enzymes in the GIT. Both approaches use peptidases from various sources such as fungi, bacteria, and germinated cereal grains (Curiel et al. 2014; Guandalini and Assiri 2014; Wolf et al. 2015; Scherf et al. 2018; Schulz et al. 2018). Postproline cutting prolyl endopeptidases from *Sphingomonas capsulata*, *Flavobacterium meningosepticum*, *Myxococcus xanthus*, *Aspergillus niger*, *Flammulina velutipes*, and *Chryseobacterium taeanense,* among others*,* have been pursued as drug candidates for the enzymatic treatment of gluten to treat celiac disease (Shan et al. 2004; Mitea et al. 2008; Schulz et al. 2018; Amador et al. 2019). Although many digestive enzyme supplements are ineffective in degrading immunogenic gluten epitopes, prolyl endopeptidase from *Aspergillus niger* is able to degrade toxic gluten epitopes (Janssen et al. 2015).

An alternative approach to alleviate the symptoms of celiac disease is to use probiotic bacteria that naturally digest gluten and specifically the toxic epitope peptides of CD. This approach could also be used to treat wheat allergies and possibly NCGS. Supplementation of probiotics in infancy was not associated with celiac disease (Uusitalo et al. 2019). It was shown by Francavilla et al. in 2017 that commercially available probiotic lactobacilli with various peptidase activities, when pooled, can hydrolase proline-rich CD epitopes of gluten and decrease gluten toxicity for CD patients (Francavilla et al. 2017).

In 2010 Helmerhorst et al. showed that gluten-degrading bacteria naturally reside in the oral cavity (Helmerhorst et al. 2010), followed by the identification of an oral microbe *Rothia aeria* that can degrade immunogenic gluten peptides (Zamakhchari et al. 2011). Several studies have shown that the human GIT, small intestinal, and colon microbiota are possibly implicated in gluten hydrolysis (Caminero et al. 2014; Herrán et al. 2017). Most of these bacteria belong to the phylum *Firmicutes*.

Why is gluten intolerance a growing concern if there are bacteria in the human GIT that can degrade harmful peptides? In contemporary Western societies, microbial diversity is decreasing with a redistributed balance (Rook 2013; Schnorr et al. 2014; Moeller et al. 2014; Mills et al. 2019). The biodiversity hypothesis states that contact with natural environments enriches the human microbiome, promotes immune balance, and protects us from allergies and inflammatory disorders (Hanski et al. 2012; Lehtimäki et al. 2018; Haahtela 2019). However, mechanisms for how microbes from the environment can colonize various sites in the body of an individual are poorly understood (Haahtela 2019).

Recently, we analysed the microbiomes of five common root vegetables—potato (*Solanum tuberosum*), carrot (*Daucus sativus*), beet (*Beta vulgaris*), neep (rutabaga) (*Brassica napus* spp. *napobrassica*), and topinambur (Jerusalem artichoke) (*Helianthus tuberosus*). We found that there is a considerable bacterial diversity in these vegetables, notably in their peels (Kõiv et al. 2019). This raises the question: Could these bacteria have an impact on gluten degradation in the human upper digestive tract? An important aspect of this concerns the ability of bacteria that we eat with raw root vegetables to resist the low pH and bile acids in the upper gastrointestinal tract. In order to provide light on these questions, we devised an experimental system: Four common root vegetables—potato (*Solanum tuberosum*), carrot (*Daucus sativus*), beet (*Beta vulgaris*), and topinambur (Jerusalem artichoke) (*Helianthus tuberosus*)—were grated and incubated in gliadin containing media at 37 °C under microoxic conditions. In addition, we studied the effects of stomach simulation (1 h, pH 3) and intestinal simulation (1 h, bile salts 0.4%). Bacteria which withstood and multiplied in these conditions were identified by 16S rRNA gene sequencing, and the amount of gliadin degraded epitopes were determined by R5 ELISA. Seven bacterial strains able to degrade gluten were isolated from vegetable samples and characterized.

## Experimental procedures

### Chemicals

Mixed gluten from wheat was obtained from Sigma (St. Louis, MO). Gliadin was made as follows: 15 g of gluten in 100 ml of 60% ethanol was shaken overnight at 37 °C and centrifuged 30 °C at 10000 rpm; the alcohol dissolved gliadin part was sterilized by filtering through a 0.22-μm syringe filter (Millex, Millipore) and used for further experiments. Synthetic immunogenic celiac gliadin-derived 33-mer peptide (LQLQPFPQPQLPYPQPQLPYPQPQLPYPQPQPF) was obtained from Pepscan, the Netherlands.

### Sampling

**Fig. 1:**
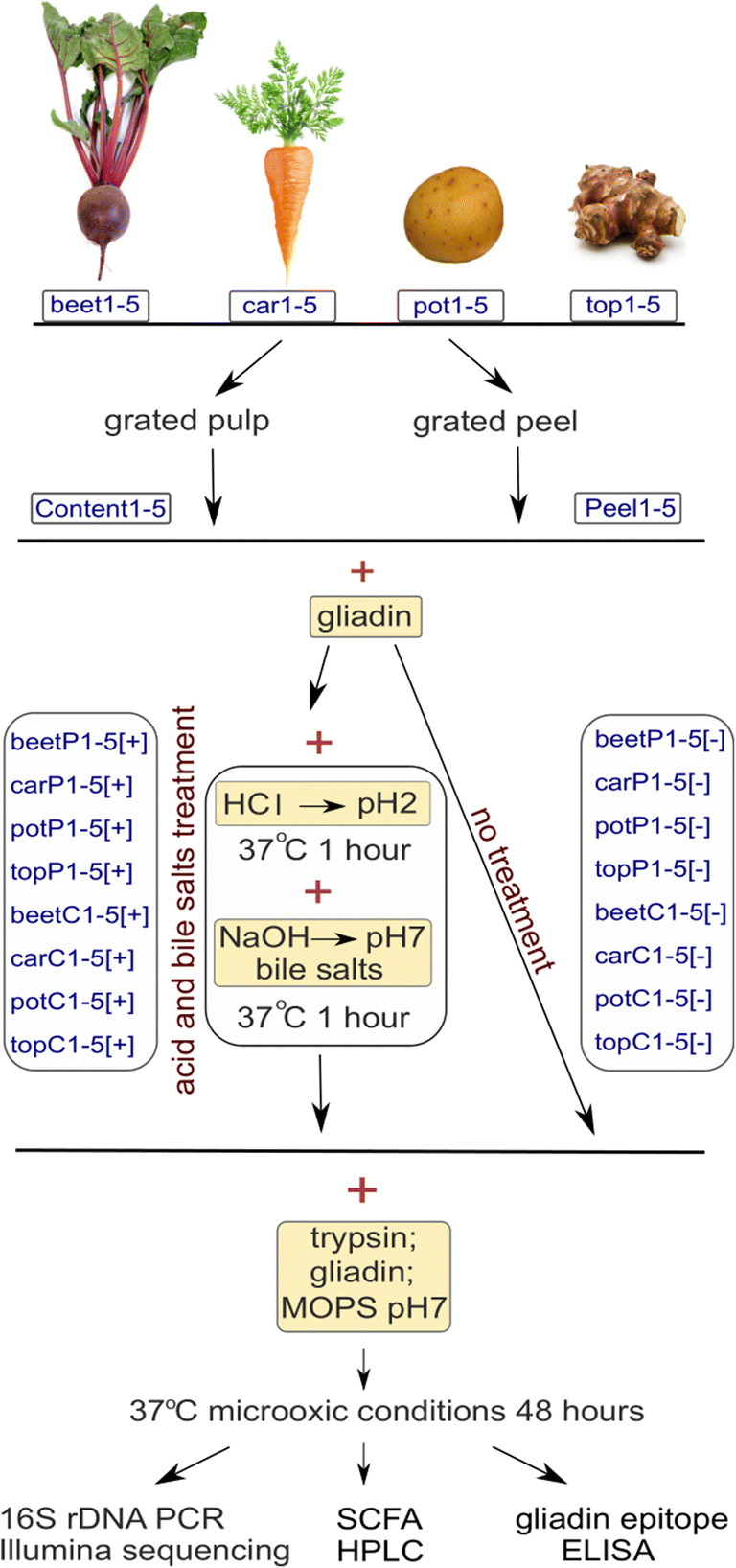
Schematic representation of the experimental strategy. Root vegetables are designated as follows: “beet,” beet; “car,” carrot; “pot,” potato; and “top,” topinambur; “C,” vegetable pulp, “P,” peel; [+] indicates acid and bile salts treatment, [−] indicates lack of treatment. SCFA, small chain fatty acids

Root vegetables carrot (*Daucus sativus*), beet (*Beta vulgaris*), and potato (*Solanum tuberosum* “Laura”) were grown by organic farming in Võnnu, Tartumaa County, Estonia (58.17 N 27.04 E). For fertilization, NPK 11-9-20, CROPCARE 11-11-21, and Allgrow® Bioplasma were used. Jerusalem artichoke (topinambur) (*Helianthus tuberosus*) was grown in virgin soil in Uhtjärve, Võrumaa, Estonia (57.89 N 26.59 E). After harvesting in October 2016, the carrots, beets, and potatoes were kept until March 2017 in a common cellar at + 5 °C. Topinambur was harvested in March 2017 freshly before the experiment. The scheme of experiment is shown on Fig. 1.

Five vegetables of similar size and shape were chosen for each species. The vegetables were carefully brushed and washed with tap water, rinsed in sterile distilled water, and thereafter kept in sterile distilled water until the next procedure. For peel extraction, the outer part of vegetable was grated with a sterile grater; 3 g of grated peel was mixed with 3 ml of gliadin (0.15%) containing sterile distilled water. This mixture was then divided equally (3 ml) into two 15-ml tubes and kept on ice until the next step. The inner tissues, i.e. the pulp of the grated vegetables, were obtained by making several cuts with a sterile scalpel to obtain an intact sample of the inner tissues. Then the pulp was grated using the same procedure as described for peels.

Next, one of these two equally divided 3-ml vegetable-gliadin mixtures was applied to HCl and bile salts (treatment [+]): pH of the sample was adjusted to 3 with 1 N HCl and incubated 1 h at 37 °C, after which pH was neutralized with 1 M NaOH. Subsequently, bile salts (final 0.4%) and trypsin (final 0.1%) were added, and this mixture was incubated for 1 h at 37 °C. During this step, trypsin (final 0.1%) was added also to the other 3-ml vegetable-gliadin mixture (control [−]) and incubated for 1 h at 37 ^o^C. The following steps were the same for both [+] and [−] samples. A 12-ml medium containing 0.07% gliadin and MOPS medium (Neidhardt et al. 1974) were added to a 3-ml vegetable mixture in order to dilute bile salts in [+] samples and support the growth of bacteria in O_2_ limiting conditions. The upper part of the 15-ml Falcon tubes free of medium were blown through with N_2_ to get rid of oxygen, after which the tubes were tightly closed with a lid and kept at 37 °C for incubation.

After 24 h of incubation, the pH was measured and neutralized with NaOH if needed. After 48 h 1.5 ml of suspension of the samples was transferred into 1.5-ml Eppendorf tubes, centrifuged for 10 min at 13,000 rpm; the pellet containing bacteria was used for DNA extraction, and the supernatant was used for both gluten content measurements and to measure the amount of small chain fatty acid (SCFA); both were stored at − 20 °C until the next procedure.

### DNA extraction, PCR, and sequencing

DNA was extracted using a RTP®Bacteria DNA Mini Kit (Stratec Biomedical Systems, Germany) according to the protocol, with one additional step: The cells were lysed by bead beating with zirconia/silica beads (BioSpec Products, USA): 0.1 mm–0.5 g with FastPrep®-24 (MP Biomedicals, USA) at 4 m/s for 3 × 60 s. The V3–V4 region of the 16S rRNA gene was amplified using primers F341ad (5′-CCAGACTCCTACGGGAGGCAG-3′) (Sakai et al. 2004) and R783ad (5′-ACCMGGGTATCTAATCCKG-3′), Phusion High-Fidelity DNA Polymerase (Thermo Fisher Scientific, USA) and approximately 20 ng of DNA in a 20-μl reaction mixture. The PCR reaction was carried out at 98 °C for 30 s followed by 20 cycles each of 98 °C for 10 s, 50 °C for 30 s, and 72 °C for 30 s, followed by 5 min at 72 °C. PCR amplifications were performed in triplicate and then pooled. The pooled PCR products were cleaned using an UltraClean PCR Clean-Up Kit (MoBio, USA), and both the quantity and quality of DNA were determined spectrophotometrically (NanoDrop 2000c). DNA sequencing was carried out using the MiSeq (Illumina PE500) (San Diego, USA).

### Sequence processing and clustering of 16S rRNA reads into operational taxonomic units (OTUs)

The total pool of sequences (2,701,997; quality filtered with Trimmomatic v 0.32 [≥Q30]) (Bolger et al. 2014) obtained from demultiplexed MiSeq reads was clustered at 97% similarity within the V3–V4 regions of 16S rRNA gene sequences into 133 nonchimeric OTUs with USEARCH tool (Edgar 2010; Edgar et al. 2011) and affiliated by using the SILVA database (version 115) with SINA aligner (Quast et al. 2012). In order to exclude sequences observed at very low frequencies, OTUs representing less than 0.001% of the total number of sequences were removed. Unclassified OTUs and OTUs with similarity to mitochondria or chloroplasts were discarded from the OTU table.

### Statistical analyses

**Fig. 2:**
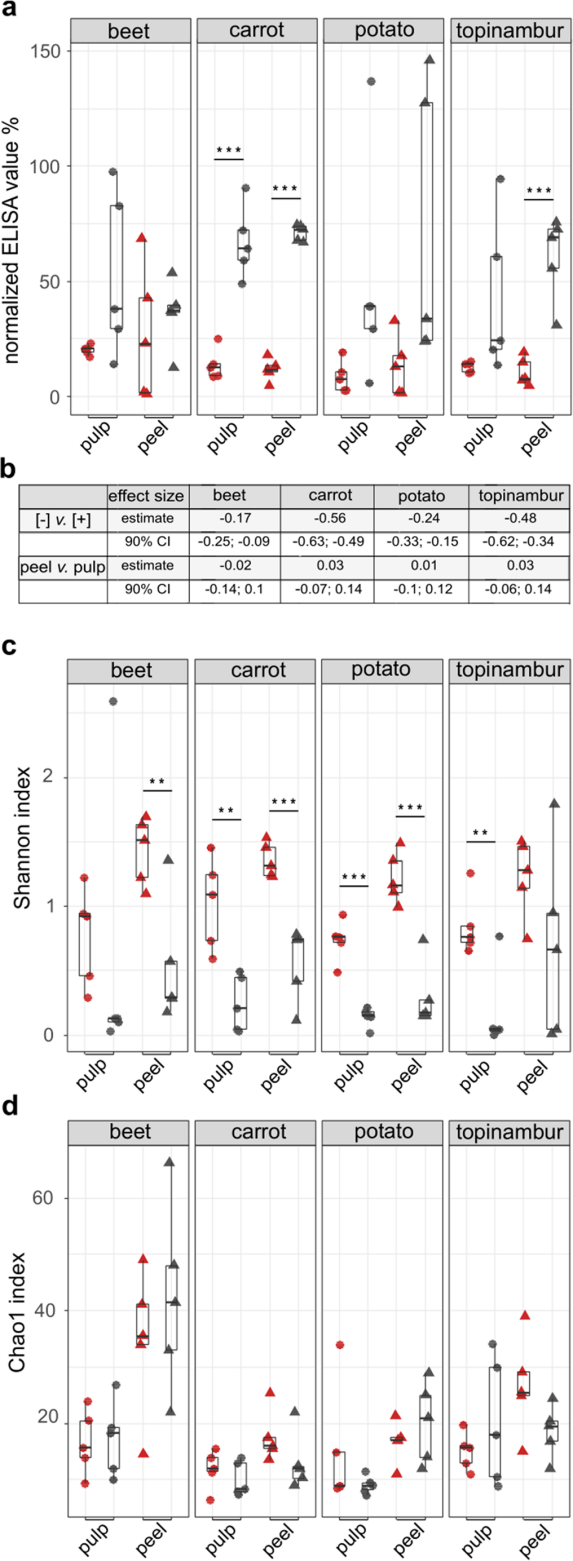
Gliadin hydrolysis and alpha-diversity of bacterial communities in medium containing one of the four grated root vegetables supplemented with gliadin. **a** Grated vegetables were incubated with gliadin for 48 h under microoxic conditions, after which the residual undigested gliadin was measured by ELISA. **b** Effect sizes ([−] vs. [+] and peel vs. pulp) with 90% CIs for each species of vegetable. **c** Microbial diversity based on the Shannon index. **d** Microbial richness based on the Chao1 index. Significant differences between acid and bile salts treatment [+] and control experiment [−] for each species of vegetable (following the Tukey post hoc test) are indicated at the top of each box: ***P < 0.001; **P < 0.01; and *P < 0.05. Grey, treated [+] samples; red, control [−] samples

Statistical analyses were performed in R 3.3.2 (R Core Team 2017). For normalization, the proportion of each OTU in unique samples was calculated. For the alpha-diversity measurement, Shannon’s Diversity and Chao1 richness were calculated with the vegan::diversity function (Jari Oksanen et al. 2017). Significant differences were determined via Tukey’s post hoc test in R (Tukey 1949). For beta-diversity measurement (Bray and Curtis 1957), a principal coordinates analysis (PCoA) was calculated with the vegdist function (Cailliez 1983). Statistical modelling for evaluating the amount of remaining gluten epitope in different samples was done using brms (Bürkner 2018) with the following model specifications: brm(value_elisa~condition + compartment + (condition|veg) + (condition|individual), data = data, family = student, prior = c(prior(normal(0, 0.2), class = b), prior(student_t(6,0.3,0.3), class = Intercept), prior(lkj(3), class = cor)), cores = 3, chains = 3, iter = 4000). Because the incorporation of compartment (pulp and core) and individual (vegetable specimen index) had a little effect on the model fit, the estimates shown in Fig. 2b are calculated from a simplified model from which those variables had been removed (loo difference 3.3 [standard error of the difference 0.9]) in favour of the simpler model: value_elisa~condition + (condition|veg).

### Gliadin quantification from growth media

The amount of gliadin in the culture media was measured with the RIDASCREEN® Gliadin competitive kit (R-Biopharm AG) according to the manufacturer’s protocol.

### Isolation of spore forming bacteria

A 150 μl of supernatant from the 48-h inoculated vegetable-gliadin mixture (see the “Sampling” section) was incubated in 50% ethanol (final concentration) for 1 h and plated on Fastidious Anaerobic Agar plate (FAA) (LAB M) containing 0.05% gliadin and on FAA plate containing 5% sheep blood (Labema Oy), which were kept under anaerobic conditions for 1 week at 37 °C. The bacteria were identified using MALDI-TOF phenotyping (Bruker Daltonik MALDI Biotyper).

### Isolation of crude extracellular bacterial proteases

*Bacillus* strains were grown in Luria Bertani (LB) broth at 37 °C with agitation for 24 h; strains from genus *Clostridium* were grown in Fastidious Anaerobic broth at 37 °C under anaerobic conditions up to 48 h. Samples were taken at 4, 6, 8, 10, 12, and 24 h of incubation: 100 μl of bacterial suspension was centrifuged at 13,000 rpm for 10 min, and the extracellular protease containing supernatant was stored at − 20 °C until later use. After collecting all samples, 10 μl of each sample was dropped on a 1.5% agar plate containing: MOPS medium (Neidhardt et al. 1974) and 0.05% gliadin. The plate was incubated at 37 °C for 2 h. The time point containing the highest amount of protease was chosen for protease production in larger amounts. For extraction of extracellular proteins, the bacterial cells were grown in a 50-ml medium as worked out at a smaller scale. The extracellular protease containing supernatant was purified and concentrated using Amicon spin columns with 10 kDa cut-off according to manufacturer’s recommendations (Amicon, USA).

### Degradation of 33-mer peptide by bacterial proteases derived from root vegetables

The peptidolytic activity of crude bacterial extracellular proteases was measured as follows: 150 μl of 50 mM MOPS buffer (pH 7), 7.5-μl 33-mer peptide (5 mg/ml), and 7.5-μl crude protease were mixed. The suspensions were incubated at 37 °C. At time intervals 0, 15, 30, 60 and 120 min, 30-μl aliquots were removed and heat inactivated for 15 min at 90 °C. Samples were centrifuged for 20 min at 13,000 rpm and analysed by UPLC and mass spectrometric identification.

### UPLC

A 15 μl of aliquots taken from 33-mer peptide incubation with crude extracellular proteases was subjected to UPLC (Waters Acquity H) BEH 130 C18 column (1.7 μm). The elution phases consisted of (A) MilliQ H_2_O containing 0.1% trifluoroacetic acid (TFA) (v/v) and (B) acetonitrile, 0.1% TFA (v/v). Peptides were eluted by using gradient: 0 to 10% buffer B 3 min, 10–70% buffer B 12 min, 70–95% B 2 min, and 95–5% B 1 min at a flow rate of 0.47 ml/min. The eluate was monitored at 214 nm. Heat inactivated (90 ^o^C for 15 min) proteins or 33-mer peptides were used as negative controls.

### Liquid chromatography tandem-mass spectrometry

All samples were purified with C18 StageTips prior to LC/MS/MS analysis (Rappsilber et al. 2007). Samples were injected to an UltiMate 3000 RSLCnano System (Dionex, California, USA) using a 0.3 × 5 mm trap column (5-μm C18 particles, Dionex) and an in-house packed (3-μm C18 particles, Dr. Maisch, Ammerbuch, Germany) analytical 50 cm × 75 μm emitter column (New Objective, Massachusetts, USA). Peptides were eluted at 250 ml/min with an A to B 8–45% 30 min gradient (buffer A 0.1% FA, buffer B 80% ACN + 0.1% FA) to a quadrupole-orbitrap Q Exactive Plus (Thermo Fisher Scientific) MS/MS via a nano-electrospray source (positive mode, spray voltage of 2.5 kV). The MS was operated with a top 10 data-dependent acquisition strategy. Briefly, one 300–1600 m/z MS scan at a resolution setting of *R* = 70,000 at 200 m/z was followed by higher-energy collisional dissociation fragmentation (normalized collision energy of 26) of the 10 most intense ions (z: + 2 to + 6) at *R* = 17,500. MS and MS/MS ion target values were 3,000,000 and 50,000 with 50 and 100 ms injection times, respectively. Dynamic exclusion was limited to 15 s. Due to the potential of peptides with small length and lack of basic amino acids, two runs were performed, one with charge state inclusion of z: + 1 to + 6, and another one with z: + 2 to + 6.

### LC/MS/MS data analysis

MS raw files were processed with the MaxQuant software package (1.6.1.0) (Cox and Mann 2008). Methionine oxidation and protein N-terminal acetylation were set as variable modifications, while cysteine carbamidomethylation was defined as a fixed modification. The search with an unspecific cleavage rule was performed against the 33-mer sequence and common human keratin contaminants. Minimal and maximal peptide lengths were set to 3 and 33 amino acids, respectively. Intensity normalization with the LFQ algorithm was enabled. False discovery rate (FDR) was kept below 1% using a target-decoy approach. All other parameters were default.

### Detection of SCFA and free sugars in growth medium

Chromatographic analyses for SCFA detection were made as described previously (Adamberg et al. 2020): Culture supernatants were filtered using AmiconR Ultra 10 K Centrifugal Filter Devices, cut-off at 3 kDa according to the manufacturer’s instructions (Millipore, United States). The concentrations of organic acids (succinate, lactate, formate, acetate, propionate, butyrate) and free sugars (mono-, di-, and trisaccharides) were determined by high performance liquid chromatography (HPLC, Alliance 2795 system, Waters, Milford, MA, United States), using Bio-Rad HPX-87H column (Hercules, CA, USA) with an isocratic elution of 0.005 M H_2_SO_4_ at a flow rate of 0.5 mL/min and at 35 °C. Refractive index (RI) (model 2414; Waters, USA) and UV (210 nm; model 2487; Waters, USA) detectors were used for quantification of the substances. The detection limit for the HPLC method was 0.1 mm.

### Sequence accession number

The sequences generated in this article are available in the NCBI (National Center of Biotechnology Information) Sequence Read Archive, and the accession ID is PRJNA629445.

The isolated strains are deposited in the Collection of Environmental and Laboratory Microbial Strains (CELMS) and financed by the Estonian Ministry of Education and Research (RLOMRCELMS); the public catalogue of which is available on the Estonian Electronic Microbial dataBase (EEMB) website http://eemb.ut.ee.

## Results

### Sampling

In order to study how much the bacteria from root vegetables (potato, beet, carrot, topinambur) impact gluten degradation in human digestive tract, we set up an experiment taking into account the fact that peels of vegetables contain a considerably higher amount and diversity of bacteria than the inner pulp and that gluten hydrolysis starts in the human mouth and the majority is hydrolysed in the duodenum. Therefore, vegetable peels and pulps were analysed separately, and all these samples were subjected to two conditions, a low pH and bile salts treatment ([+]), and to a no-treatment control ([−]). Both [+] and [−] samples were kept at human body temperature in microoxic conditions (Fig. 1). All samples were treated with trypsin in order to solubilize gliadin, which precipitates in water-based solutions. The media in which the bacteria were grown contained 1.5% grated vegetable under study, 0.07% gliadin, and MOPS medium. In this medium, the main carbon source originated from the particular vegetable, MOPS medium was added to ensure neutral pH, and gliadin could be used as nitrogen source, if degraded. The measurement points were taken 48 h after the start of the experiment.

After incubating the samples for 24 h at 37 °C under microoxic conditions, the pH was measured. It turned out that pH had dropped from 7 to ~ 5 in all carrot samples and in topinambur and beet [−] samples. In these samples, the low pH was neutralized with NaOH. In all potato samples, the pH stayed neutral. After 48 h of incubation, the change of pH was similar to that at 24 h: the pH has dropped from 7 to ~ 5 in all carrot samples , in topinampur and beet [−] samples, and in some topinambur peel [+] samples. The pH stayed neutral in all potato samples.

The growth of bacteria was assessed by visual inspection because it was not possible to measure OD due to the background of plant debris. The [−] samples were visibly more absorbent than [+] samples. It was also possible to observe intensive growth of bacteria in [−] samples based on their strong smell. One sample, beetC3[+], had no visible growth.

A 1.5 ml of the culture was taken and divided into two parts after centrifugation: cells for DNA isolation and supernatant for gliadin and SCFA detection.

The concentration of isolated bacterial DNA fluctuated accordingly to the growth of bacteria in the samples: The amount of DNA was smaller from [+] samples than from [−] samples.

### Degradation of gliadin epitope by vegetable-originated bacteria

The immunogenic gliadin-derived peptides remaining after the 48 h [+] and control [−] samples were quantified by RIDASCREEN® Gliadin competitive kit. The R5 monoclonal antibody recognizes the gluten epitopes QQPFP, QQQFP, LQPFP, QLPFP, and related sequences. These gluten epitopes were at higher levels in samples that had passed through acid and bile salts treatment [+] than in control samples [−] (Fig. 2a). In pulp and peel [−] samples of carrot and in peel [−] samples of topinambur had significantly lower amount of gluten epitopes left than in respective [+] samples (Tukey post hoc test *P* < 0.001). Our robust linear model suggests, on average, an ~ 20 percentage point reduction in gliadin hydrolysis activity upon [+] treatment for beet and potato and an ~ 50 percentage point reduction for carrot and topinambur (Fig. 2a,b).

There was a slight increase of the signal in potato containing samples, suggesting that components of the vegetables interfere with the antibody reaction. It has been shown that starch influences the accessibility of gluten epitopes (Smith et al. 2015).

### Gluten-degrading bacteria in vegetable-based samples

#### The microbial composition

There are several challenges for vegetable-derived bacteria to survive in the human gastrointestinal system. Firstly, most of the plant endophytes do not grow well at 37 °C under anaerobic conditions. Secondly, conditions in the stomach are expected to kill most bacteria. We studied the microbial composition of the bacterial communities grown in microoxic environment at 37 °C and treated with HCl and bile salts.

All 80 samples were analysed by sequencing the V3–V4 region of 16S rRNA gene fragments. In total, 1,460,685 high quality reads were obtained and assigned to 121 operational taxonomic units (OTUs). The microbial diversity of OTUs calculated by the Shannon index was higher in [−] samples, whereas the estimated richness (Chao1) was rather uniform in both treated [+] and control [−] samples (Fig. 2c,d). This shows that acid and bile salts treatment causes dominance of specific bacterial groups, although minor OTUs remain detectable. We have previously shown that there are large differences in the bacterial diversity between peel and pulp samples (Kõiv et al. 2019). Incubation under anoxic conditions at 37 °C abolished this difference independent of acid and bile salts treatment.

**Fig. 3:**
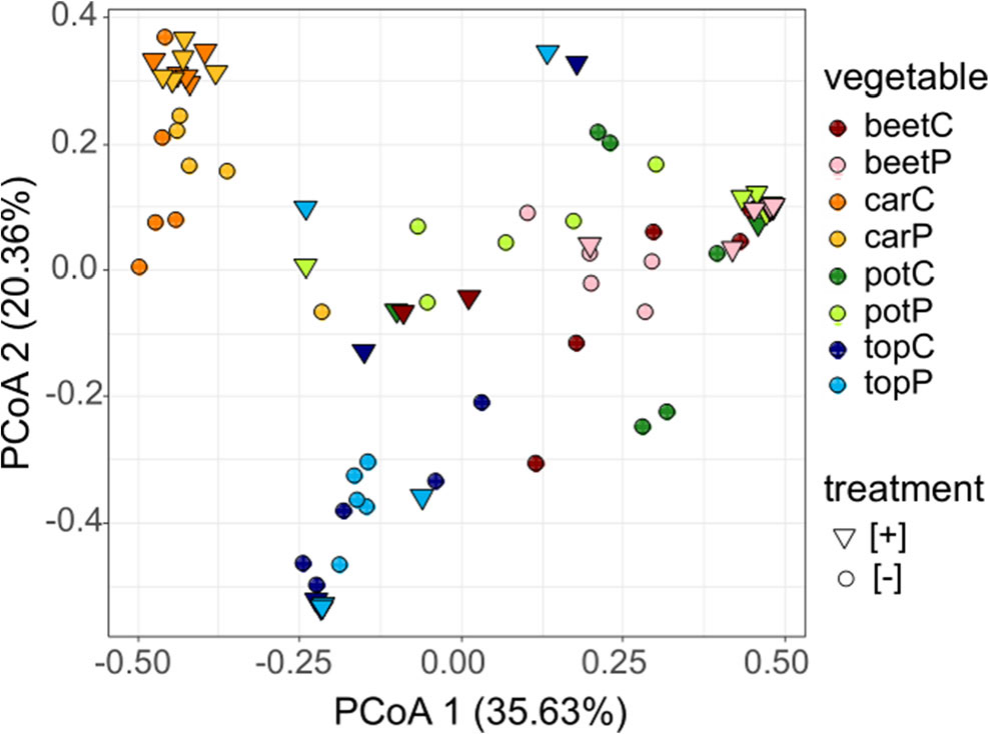
Bacterial community structure in medium containing one of the four grated root vegetables supplemented with gliadin**.** A principal coordinates analysis (PCoA) plot of Bray-Curtis dissimilarity of all OTUs defined in the samples of root vegetables was incubated for 48 h in microoxic conditions at 37 °C. For sample names, see Fig. 1

The similarity of the bacterial communities between different vegetable samples was examined using the principal coordinates analysis (PCoA). Clustering of the samples occurred by vegetable species rather than by treatment (Fig. 3). Carrot and to some extent also topinambur samples were separated on PCoA. Potato [+] and some beet [+] samples clustered together, but most of the beet [−] and potato [−] samples did not form distinct clusters. There was no clear clustering between peel and pulp in the microbiota studied.

**Fig. 4:**
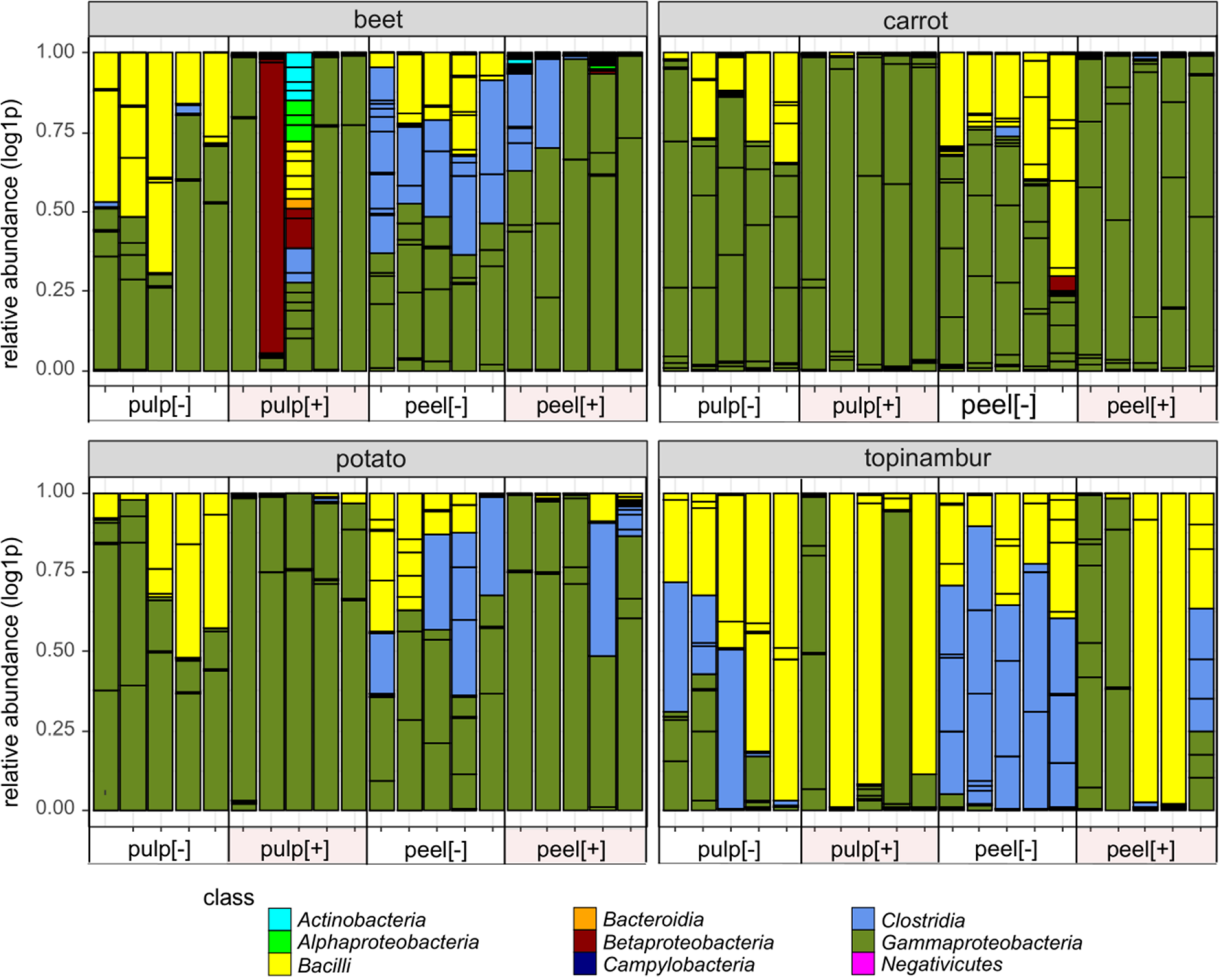
Relative sequence abundances of bacterial classes found in medium containing one of the four grated root vegetables supplemented with gliadin. All OTUs defined in the samples of root vegetables incubated for 48 h under microoxic conditions at 37 °C are indicated on the bar based on a class colour code. For sample names, see Fig. 1

Five phyla, *Actinobacteria*, *Firmicutes*, *Proteobacteria*, *Bacteroidetes*, and *Epsilonbacteraeota*, represented by classes *Actinobacteria*, *Acidobacteria*, *Bacilli*, *Clostridia*, *Negativicutes*, *Alphaproteobacteria*, *Betaproteobacteria*, *Gammaproteobacteria*, *Bacteroidia*, *Campylobacteria*, and *Acidimicrobiia* were detected in at least in one sample (Fig. 4). The most abundant class of bacteria/OTU belong to class *Gammaproteobacteria* that dominate exclusively in [+] samples of carrot and in [+] samples of potato pulp. The other abundant group of bacteria/OTUs belong to phylum *Firmicutes*, represented by classes *Bacilli* and *Clostridia.* These OTUs are more abundant in samples that have not passed the acid and bile salts treatment.

**Fig. 5:**
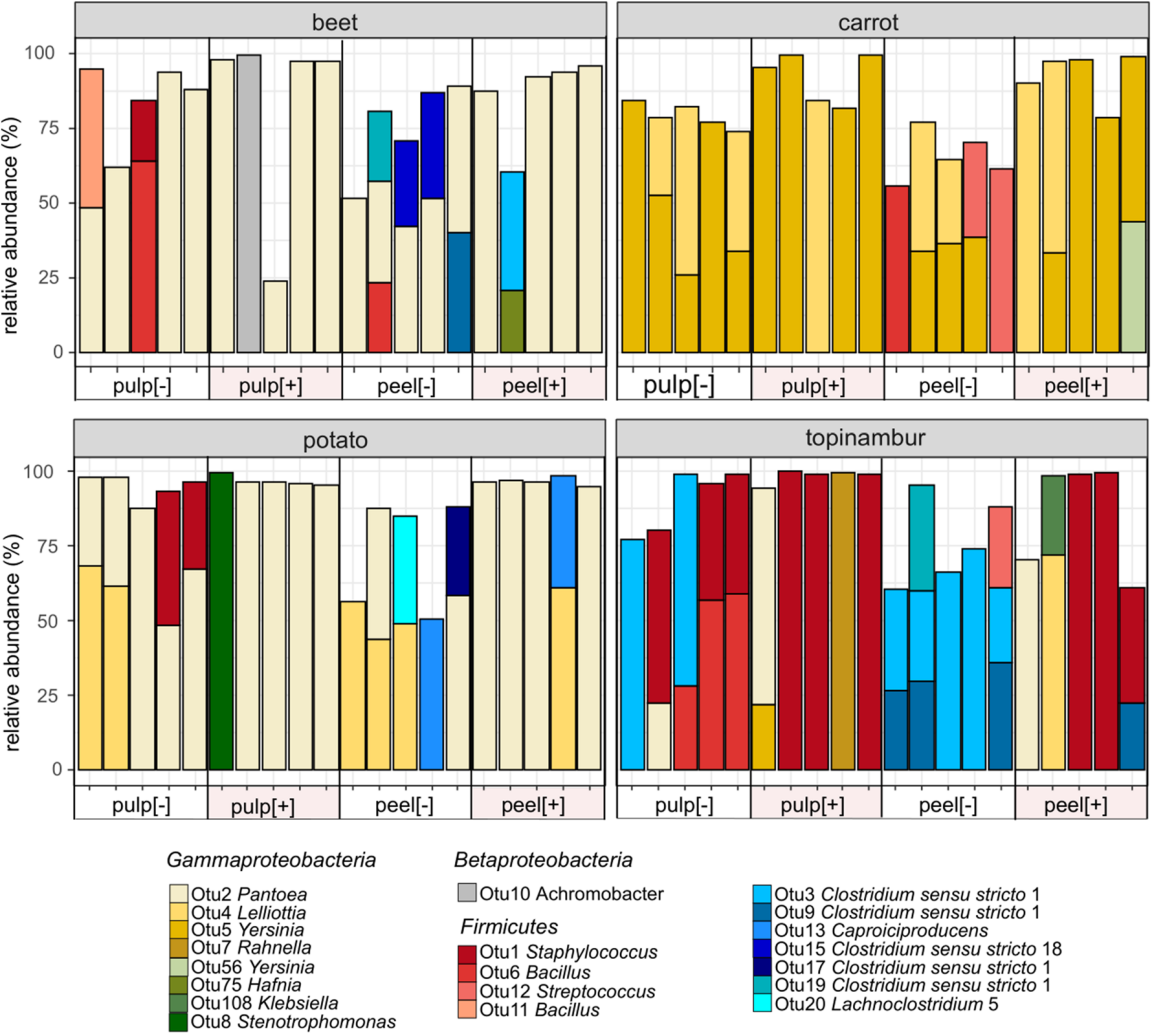
Dominant OTUs defined in medium containing one of the grated four root vegetables supplemented with gliadin. The samples of grated root vegetables were incubated for 48 h in microoxic conditions at 37 °C. OTUs whose relative abundance was more than 20% of total abundance of the sample are indicated. For sample names, see Fig. 1

In order to examine the dominant OTUs, all OTUs whose relative abundance was more than 20% of the total abundance in the sample are shown in Fig. 5. In the majority of [+] samples, only one OTU took over more than 80% the culture, the bacterial culture in [−] samples is more complex, but still 50–75% of the population consists of two different OTUs (Fig. 5). The emergence of several dominant OTUs in [−] samples is in line with the increased Shannon index. The PCoA ordination can be explained by looking at the dominant OTUs: OTU5 *Yersinia* discriminates carrot from other vegetable samples. Topinambur is discriminated from other vegetable samples by members of phylum *Firmicutes*: in case of [+] samples by *Bacilli* (OTU1 *Staphylococcus*) and in case of [−] samples by *Clostridia* (OTU3 *Clostridium* sensu stricto 1 and OTU9 *Clostridium* sensu stricto 1) and *Bacilli* (OTU1 *Staphylococcus* and OTU6 *Bacillus*). The dominant OTU in both beet and potato samples is OTU2 *Pantoea,* which explains the poor PCoA clustering of the OTUs that originate from these vegetables (Figs. 5 and 3).

Some bacterial groups in the *Enterobacteriaceae* family, OTU2 *Pantoea* and OTU5 *Yersinia*, survive under acid and bile salts treatment and are predominantly found in potato, beet, and carrot [+] samples. In topinambur, *Staphylococcus* OTU1 also successfully passed acid and bile salts treatment.

As mentioned above, we observed a strong smell from control [−] samples, which indicates production of SCFA, particularly butyrate. We analysed production of SCFA and consumption of main mono- and disaccharides derived from vegetables in samples. We observed that the members of order *Clostridiales* have a significant correlation with butyrate production (Supplementary Materials (Text S1, Fig. S1)).

#### Proteases from vegetable-originated bacteria

Because bacteria from the *genera Bacillus* and *Clostridia* that can potentially produce extracellular protease are spore forming, we isolated spores from samples that had the least gliadin left after 48 h of incubation.

**Fig. 6:**
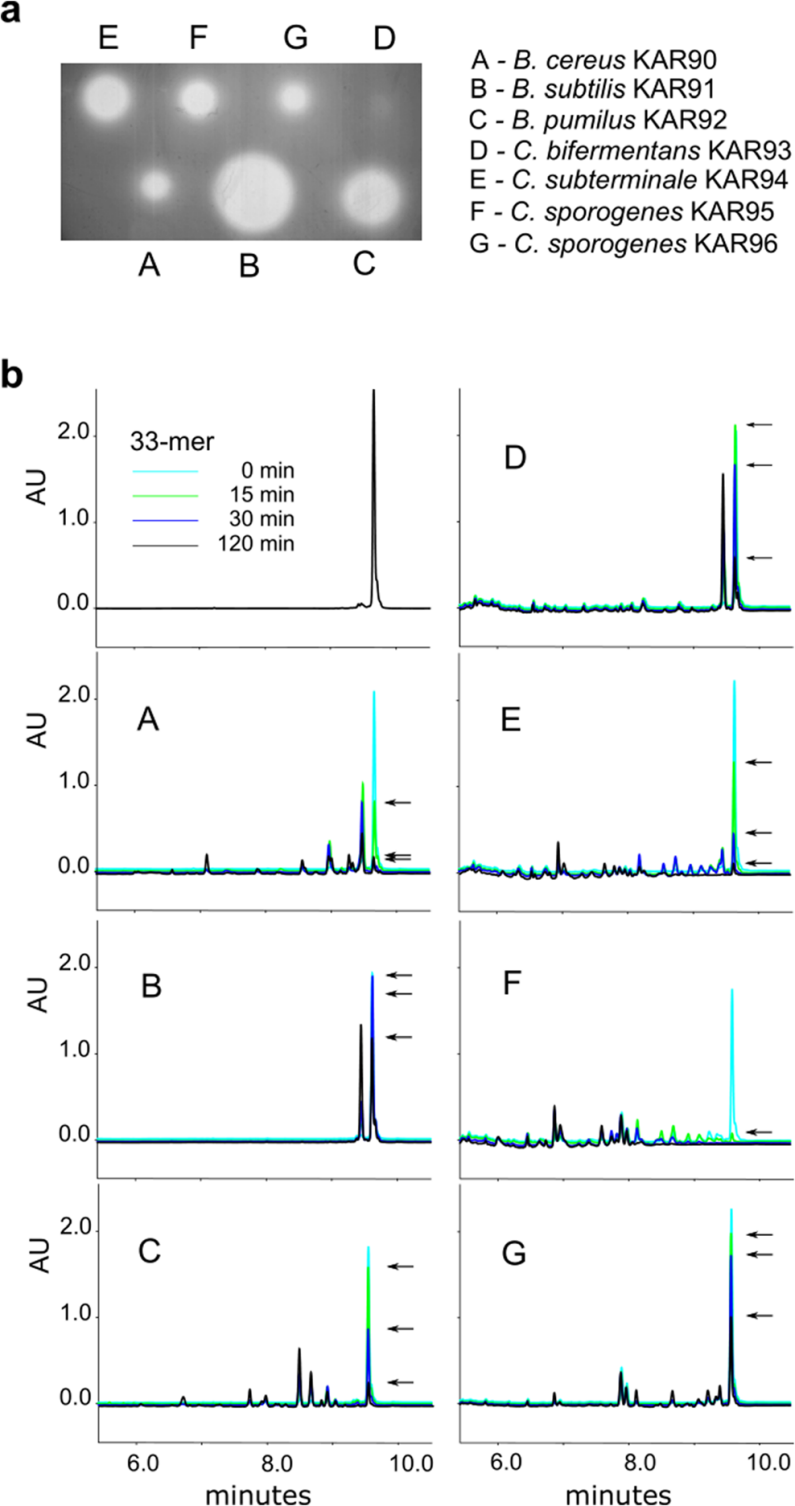
Glutenase activity of extracellular proteases produced by bacteria originated from root vegetables. **a** Two μl extracellular protease extracts pipetted onto gliadin (0.05%) agarose plate and incubated for 2 h. **b** UPLC chromatograms of 33-mer peptide hydrolysis products. A 33-mer peptide (final concentration 250 μg/ml) was incubated for 120 min at 37 °C with extracellular protease extracts. Arrows point to the original uncleaved 33-mer peptide

**Fig. 7:**
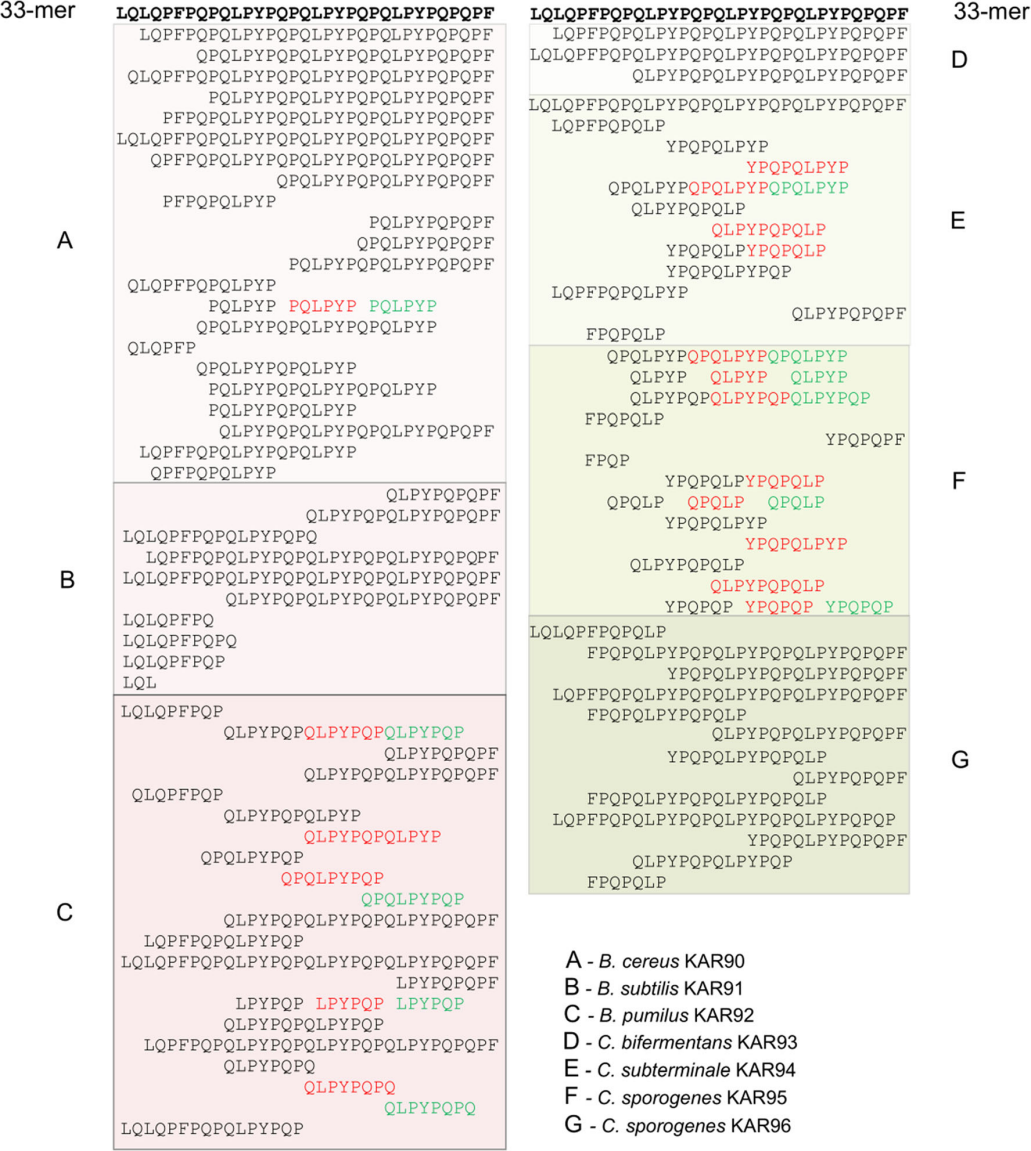
Peptides generated after cleavage of 33-mer peptide by extracellular proteases produced by bacteria originated from root vegetables. The 33-mer peptide fragments were identified by mass spectrometry. Fragments are shown in descending order based on LFQ (label-free quantification) intensity value. LFQ intensity value 10^10^ was taken as threshold. Different colours indicate the repetitive peptide sequences of the 33-mer peptide

These spores were plated on FAA + gliadin plates and also on an FAA + blood plate to provide a better chance for germination. After 5 days of cultivation under strict anaerobic conditions at 37 °C, we streaked all colonies that emerged onto FAA + gliadin plates. The gliadin-degrading bacteria were detected only on control [−] samples. Bacteria with a hydrolytic halo surrounding the colony were identified as *Bacillus pumilus* (beetP3[−]), *Bacillus cereus* (beetP4[−], carP4[−], potP4[−]), *Bacillus subtilis* (carC5[−], beetP5[−]), *Bacillus circulans* (potP4[−]), *Bacillus licheniformis* (beetP5[−]), *Bacillus psychrosaccharolyticus* (topP1[−]), *Clostridium bifermentans* (potP1[−]), *Clostridium sporogenes* (beetP1[−], topP1[−]), and *Clostridium subterminale* (potP4[−], potP5[−]). Three *Bacillus* strains and four *Clostridium* strains that produced the largest hydrolytic halos were cultivated in liquid medium to assess their protease production. Two μl of concentrated crude protease solutions were pipetted onto a gliadin-containing agarose plate (Fig. 6a). The halos produced by different proteases varied greatly in size. We used the same crude protease solutions to find regions of gliadin cleavage. We deployed human protease-resistant immunogenic gliadin 33-mer peptide, which is widely used to study the cleavage pattern of proline endopeptidases (Shan et al. 2005). This 33-mer peptide encompasses 6 overlapping immunogenic 9-mer human T cell epitopes: PQPQLPYPQ (3 copies), PYPQPQLPY (2 copies), and PFPQPQLPY. After incubation of the 33-mer peptide with protease(s), the peptide fragments generated were studied by UPLC and mass spectrophotometry (Figs. 6b and 7). All proteases under investigation have cutting sites within the 33-mer peptide. *B. subtilis* KAR91 (originated from beetP5[−]) and *C. bifermentans* KAR93 (originated from potP1[−]) proteases have the same cleavage pattern in 33-mer, degrading short regions (2–8 amino acids) from the N-terminus while leaving the immunogenic peptide region intact. *B. cereus* KAR90 (originated from potP4[−]) protease/proteases possess mostly N-terminal exopeptidase activity but could also have some other type of proteases based on the UPLC and mass spectrophotometry results. Proteases from the other bacteria studied, i.e. *B. pumilus* KAR92 (originated from beetP3[−]), *C. subterminale* KAR94 (originated from potP5[−]), *C. sporogenes* KAR95 (originated from beetP1[−]) and *C. sporogenes* KAR96 (originated from topP1[−]), cut after proline, as expected for a proline endopeptidase. In addition, the proteases from *B. pumilus* KAR92, *C. sporogen*es KAR96, and *C. subterminale* KAR94 seem to have some additional peptidase activity. The highest endopeptidase activity is conveyed by *C. sporogenes* KAR95. Although not very efficient in hydrolyzing larger molecules of gliadin (Fig. 6a), it completely destroyed the 33-mer peptide during the 30 min of incubation (Figs. 6b and 7).

In conclusion, bacteria of vegetable origin from the genera *Bacillus* and *Clostridium* produce variable extracellular proteases that can efficiently hydrolase gliadin, and some of these proteases can destruct proline-rich peptides that are hardly digested by human proteolytic enzymes.

## Discussion

Gluten intolerance has been an emerging problem over the last 20 years. At the same time, knowledge about the impact of bacteria and other microorganisms on gluten degradation has increased (Shan et al. 2004; Stepniak et al. 2006; Zamakhchari et al. 2011). It has been shown that the human digestive tract has bacteria that can successfully break down and eliminate peptide fragments that are resistant to human peptidases (Caminero et al. 2014; Herrán et al. 2017). Extracellular protease genes are abundant in free-living bacteria (Nguyen et al. 2019), and the most powerful proteases that degrade gluten are produced by microorganisms isolated from the surrounding environment (Mitea et al. 2008; Amador et al. 2019). Knowing that the diversity of human microbiome is decreasing as more people move away from nature (Grönroos et al. 2019), it is tempting to hypothesize that there is a direct connection between gluten intolerance and a decrease in the available microorganisms capable of gluten destruction.

This study aimed to highlight the process of bacterial transfer from nature to the human digestive tract and describe the capacity of bacteria to degrade gliadin. We demonstrate that bacteria that reside in root vegetables (beet, carrot, potato, topinambur) possess strong gluten/gliadin hydrolysing capabilities.

Using a model system, we show that the bacteria that can multiply under conditions similar to the human gastrointestinal tract (anaerobiosis, 37 °C) mostly belong to the family *Enterobacteriaceae* and families *Bacillaceae* and *Clostridiaceae* (Figs. 4 and 5)*.* Another recent study of ours indicates that *Enterobacteriaceae* is one of the most abundant groups of endophytes in the pulps of vegetables (Kõiv et al. 2019). The most diverse groups of plant endophytes belonging to the phylum *Actinobacteria*, classes *Alfa*- and *Betaproteobacteria*, and the order *Pseudomonadales,* apparently did not resist anoxic conditions at 37 °C and/or were outcompeted by bacteria that are more adapted to these conditions. This does not exclude the possibility that these bacteria stay in mouth for shorter periods and contribute to gluten hydrolysis. In 2017, Tian et al. demonstrated that endogenous salivary microbes produce proteases, but their activities are incomplete and liberate peptides from larger gluten proteins that ultimately reach the small intestine and can cause CD (Tian et al. 2017).

*Enterobacteriaceae*, genera *Pantoea, Lelliottia,* and *Yersinia* seem to resist low pH and bile acids better than members of *phylum Firmicutes,* genera *Bacillus* and *Clostridium* (Figs. 4 and 5). Which is surprizing because the bacteria from the latter genera can form spores that can more effectively resist the harsh conditions of the GIT. One possible explanation is that in the vegetables we studied, the bacteria were in a vegetative stage or, alternatively, there were more *Enterobacteriaceae* in the starting culture.

Gliadin was more efficiently digested in samples not treated with acid and bile salts. However, we do not know which had a higher impact on gliadin degradation, the total number of bacteria or the change in the species composition. In addition, a higher diversity could facilitate cometabolism. It has been shown that ten *Lactobacillus* strains in a pool, but not independently, can completely destroy toxic gliadin epitopes (Francavilla et al. 2017). Similar co-degradation has been shown for immunogenic peptides produced by *Pseudomonas aeruginosa* that can be degraded to non-immunogenic peptides by *Lactobacillus* spp. (Caminero et al. 2014).

In most potato, beet, and carrot samples, it is possible to hypothesize which bacteria were responsible for gliadin degradation: most prominently *Bacillus* and *Clostridium* sensu stricto 1. *Bacillus* and *Clostridium* species/strains produce variable extracellular proteases with different target specificity. These proteases are secreted into culture media. If there are proteases with different specificity in the culture medium, gliadin could easily be degraded into shorter peptides and amino acids that can be used by other bacteria that do not produce any protease. Both *Bacillus* and *Clostridium* are very versatile groups of bacteria, the species affiliation giving only hints about possible protease production and specificity. Therefore, we studied the degradation of a 33-mer gliadin peptide by crude extracellular proteases more closely. Proteases from four bacteria, two *Clostridium sporogenes* strains KAR95 and KAR96, *Clostridium subterminale* KAR94, and *Bacillus pumilus* KAR92, have similar cleavage sites in the 33-mer peptide: They hydrolyse the peptide at the carboxy terminus of the internal proline residues—the typical cleavage pattern of postproline endoproteases (Fig. 7). The most efficient degradation of the 33-mer peptide was carried out by *Clostridium sporogenes* KAR95; however, the degradation of whole gliadin protein was quite low (Fig. 6a,b). This indicates that *Clostridium sporogenes* KAR95 produces mostly prolyl endopeptidase. The activity of prolyl endopeptidase is often restricted to substrates shorter than 30 amino acids and hydrolysis at a central position (Mika et al. 2015). In conclusion, bacterial strains that originate from vegetable roots produce proteases with very different efficiency and target specificity.

Most surprisingly, the bacterial species that display extracellular proteolytic activity isolated from human faeces (*Bacillus licheniformis*, *B. subtilis*, *B. pumilus*, *Bifidobacterium longum*, *Clostridium sordellii*, *C. perfringens*, *C. botulinum/sporogenes*, *C. butyricum/beijerinckii*, *Enterococcus faecalis*, *E. faecium*, *Propionibacterium acnes*, *Pediococcus acidilactici*, *Paenibacillus jamilae*, *Staphylococcus epidermidis*, *S. hominis*, and *Stenotrophomonas maltophilia*) (Caminero et al. 2014) are largely the same bacteria we found in root vegetables. *Clostridium sporogenes* and *Bacillus pumilus* isolated from human faeces has been shown to degrade the 33-mer with high activity (Caminero et al. 2014). The majority of bacteria in the human GIT that can degrade gluten belong to phylum *Firmicutes*: lactobacilli and bacilli in the upper part of the duodenum, where the degradation of proteins by human enzymes take place (Nistal et al. 2016; Herrán et al. 2017), and clostridia, mostly in the colon (Caminero et al. 2014). Although lactobacilli are well-known probiotics, they are less prominent in protein degradation than bacilli and clostridia (Bergey 2005). *Lactobacilli* can hydrolase shorter peptides (Francavilla et al. 2017); however, bacilli and clostridia can destruct also larger molecules. The degradation products have to be carefully considered as it has been shown that proteases produced by some bacteria increase the immunogenicity of gluten peptides (Nistal et al. 2016; Caminero et al. 2019).

In addition to possible impact on gluten degradation, we observed a correlation between the production of butyrate and the presence of members of the family *Clostridiaceae* (Fig. S1). Butyrate plays a key role in maintaining human gut health, is a major source of energy to the colonic mucosa, and is also an important regulator of gene expression, inflammation, differentiation, and apoptosis in host cells (Louis and Flint 2009).

Members of the order *Clostridiales* are not frequently found in plants (Hacquard et al. 2015). Under enrichment conditions (microoxic conditions and 37 °C) applied in our experiment, the diversity and abundance of different OTUs assigned to the order *Clostridiales* increased remarkably (Figs. 4 and 5; Fig. S1). It has been shown that *Clostridial* species are protective in the development of food allergies (Blázquez and Berin 2017; Abdel-Gadir et al. 2019). Colonization of germ-free mice with a consortia of commensal *Clostridia* species induces regulatory T cells and protects against food allergies (Atarashi et al. 2013; Stefka et al. 2014). In 2014, Seedorf et al. showed that clostridia from soil can colonize and persist in mouse gut (Seedorf et al. 2014). We do not know how well environmental clostridia can invade and persist in the human gut, but this study demonstrates that together with raw root vegetables, we swallow a variety of bacteria that could reach the colon and contribute to the function of our immune system.

Therefore, we hypothesize that increasing the amount of raw vegetables in the diet might increase the diversity of bacteria in human GIT and thereby alleviate the symptoms of gluten related diseases. Extensive dietary studies are needed to test this hypothesis.

## Electronic supplementary material

ESM 1 (PDF 456 kb)

## Data Availability

(Data transparency)
