# Supplementary material for: Microbiome of root vegetables—a source of gluten-degrading bacteria
Source: Appl Microbiol Biotechnol. 2020 Sep 2;104(20):8871–85. doi: 10.1007/s00253-020-10852-0 (PMC7502452; doi:10.1007/s00253-020-10852-0)

SUPPLEMENTARY MATERIAL

Microbiome of root vegetables – a source of gluten degrading bacteria

Viia Kõiv[1]*, Kaarel Adamberg[2], Signe Adamberg[2], Ingrid Sumera[2], Sergo Kasvandik[1], Veljo Kisand[1], Ülo Maiväli[1], Tanel Tenson[1]

[1]Institute of Technology, University of Tartu, Tartu, Estonia

[2] School of Science: Department of Chemistry and Biotechnology, Tallinn University of Technology, Tallinn, Estonia

***Corresponding author:**

Viia Kõiv

E-mail: viia.koiv@ut.ee

Tel: +372 555 44 353

Content

Supplementary Text 1

Production of SCFA

We observed a strong smell from control [-] samples (not treated with acid and bile salts), which indicates production of SCFA, particularly butyrate. Thus, the concentrations of SCFA and the main mono-and disaccharides derived from vegetables were studied in a subset of samples. High amounts of free sugars (glucose, fructose and sucrose) have been consumed in beet and carrot samples after 48 hours of incubation. At the same time, accumulation of SCFA-s was observed (Fig. S1a). In one sample, beetC2[+], higher amount of free sugars was left over with a lower production of SCFA-s.

In both potato and topinampur, in which sugars are mainly in polymeric form, starch and inulin respectively, the amount of free sugars is low. In the case of topinambur, the amount of free sugars rises in some samples after 48 hours of incubation, compared to the start point. This points to the degradation of inulin which results in an accumulation of fructose monomers (Fig. S1a). In potato, the amount of free sugars is very low before incubation and disappears completely in 48 hours. One exception is sample potC1[+] with higher amount of free sugars left, and almost no SCFA was produced in this sample either (Fig. S1a). The low amount of free sugars after 48 hours of incubation suggest that these samples lack bacteria with extracellular amylase activity. This explains the relatively stable pH of the potato incubation medium compared to other vegetables (chapter "Sampling").

The production of acetate, formiate and lactate is quite evenly distributed between different vegetable samples, however, butyrate is produced only in some samples of topinambur and beet (Fig. S1a). In order to find out which bacteria could be butyrate producers, we calculated the Pearson correlation coefficient between the production of butyrate and OTU abundances at the level of order. Only *Clostridiales* has a significant correlation with butyrate production (r=0.86). In this order, two families, *Clostridiaceae* (r=0.92) and *Peptostreptococcaceae* (r=0.68) correlate with butyrate production. One OTU from each family significantly correlates with butyrate production: OTU3 *Clostridium sensu stricto* 1 from *Clostridiaceae* (r=85) and OTU14 *Paeniclostridium sp*. from *Peptostreptococcaceae* (r=68) (Fig. S1b).

Fig. S1 Production of SCFA by bacteria derived from four root vegetables. **a** Amount of free sugars and SCFA in medium after incubating the grated vegetables with gliadin for 48 hours in anaerobic conditions at 37ºC. Acid and bile salts treatment [+] and control [-] are indicated. **b** Association based on Pearson's correlation coefficient (r) between OTU abundance and production of butyrate. For sample names see Fig. 1

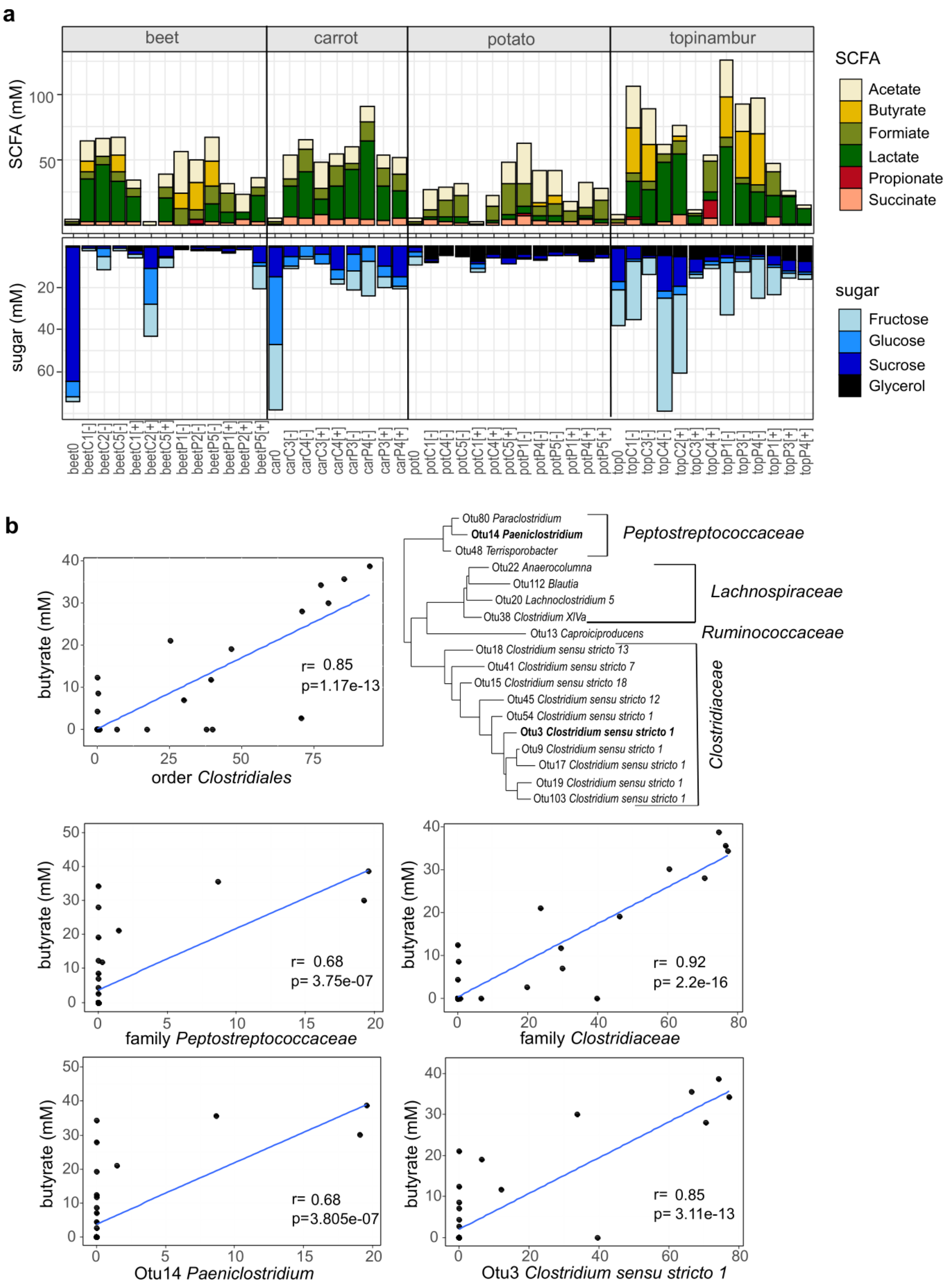

Supplement: Supplementary file 1 — (PDF 456 kb) [file 253_2020_10852_MOESM1_ESM.pdf]
